# Primary Causes and Direct Medical Cost of Heart Failure Among Adults Admitted with Acute Decompensated Heart Failure in a Public Tertiary Hospital, Kenya

**DOI:** 10.5334/gh.1426

**Published:** 2025-05-02

**Authors:** Victor M. Wauye, G. Titus Ngeno, Chrispine O. Oduor, Felix A. Barasa

**Affiliations:** 1 Department of Internal Medicine, Moi University, Eldoret, Kenya; 2 Division of Cardiology, Department of Medicine, Duke University, Durham, USA; 3 Department of Cardiology, Moi Teaching and Referral Hospital, Eldoret, Kenya

**Keywords:** Primary causes, heart failure, direct medical cost, hospitalization, Moi Teaching and Referral Hospital

## Abstract

**Background:**

Heart failure (HF) is a major contributor to cardiovascular morbidity and mortality. Adversely impacting health outcomes in Kenya and other developing countries, data on the direct medical cost of HF hospitalization remain limited.

**Methods:**

This was a prospective study conducted at Moi Teaching and Referral Hospital. Patients with HF were identified by sequential medical chart abstraction. Primary causes were extracted from echocardiogram reports and adjudicated by a cardiologist. Direct medical cost of hospitalization was derived using activity-based and micro-costing methods, adopting payers’ system perspective. Drivers of overall cost were explored using linear regression models.

**Results:**

142 participants were consecutively recruited from September to November 2022. 51.4% were females, and the mean age was 54 (SD 20). The leading primary causes were cor pulmonale (CP), 28.9%; dilated cardiomyopathy (DCM), 26.1%; rheumatic heart disease (RHD), 19.7%; hypertensive heart disease (HHD), 16.9%; ischaemic heart disease (IHD), 6.3%; and pericardial disease (PD), 2.1%. Overall direct cost of HF hospitalization was KES 11,470.94 (SD 8,289.57) [USD 93.49 (67.56)] per patient per day, with the mean length of hospital stay of 10.1 (SD 7.1). RHD incurred the highest costs, KES 15,299.08 (SD 13,196.89) [USD 124.70 (107.56)] per patient per day; IHD, KES 12,966.47 (SD 6656.49) [USD 105.68 (54.25)]; and DCM, KES 12,268.08 (SD 7,816.12) [USD 99.99 (63.71)]. The cost of medications was the leading driver, β = 0.56 (0.55 – 0.56), followed by inpatient fees, β = 0.27 (0.27 – 0.28), and laboratory investigations, β = 0.19 (0.18 – 0.19).

**Conclusion:**

Cor pulmonale, CM, RHD, and HHD were the major causes of HF. The overall direct medical cost of hospitalization was extremely expensive compared with the Kenyan average monthly household income per capita. Widespread comprehensive health insurance coverage is therefore recommended to cushion families against such catastrophic health expenditures besides public health measures aimed at addressing primary causes of HF.

## Introduction

Cardiovascular diseases (CVDs) are the most common cause of global morbidity and mortality, accounting for more than 17.9 million deaths annually in the recent years. More than 75% of these deaths occur in the middle- and low-income countries (1). Heart failure (HF) is one of the most common primary CVD diagnoses among hospitalized medical patients in Sub-Saharan Africa (SSA) (2,3). Further, the main causes of HF in SSA have been traditionally described as hypertensive heart disease (HHD), dilated cardiomyopathy (DCM), rheumatic heart disease (RHD), and less commonly, other causes of HF such as ischemic and congenital cardiomyopathies (4,5).

Treatment of HF confers a significant financial burden both to the healthcare system and to the affected families, being largely driven by direct medical costs of hospitalization (6,7). Direct medical costs accounted for about 60% of the total costs in a review by Cook et al. (6). Data on the cost of treatment of HF in Africa is, however, still limited, with one study having reported USD 2,128 per patient per year (8).

To the best of our knowledge, there is no published study on the medical cost of hospitalization for HF in Kenya despite the efforts towards the achievement of universal health coverage. According to the 2023 Kenya Demographic Health Survey, healthcare expenditure in Kenya is mainly paid for out of pocket, with only 26.5% of the Kenyans having any form of health insurance (9). Of these, only 24% are covered by the government-sponsored National Health Insurance Fund (NHIF), while the rest are covered by private insurance. As of November, 2023, following enactment of the Kenya Social Health Insurance Act, NHIF was repealed and replaced by a new Social Health Authority. Considerably, only 35% of hospitalized patients had NHIF cover at tertiary government health facilities (10). Furthermore, it is notable that about 83% of the workforce in Kenya is comprised of the informal sector (11). A study assessing the uptake of NHIF among informal workers in Western Kenya showed that only 12% had health insurance coverage, with the majority experiencing catastrophic health expenditure (12), meaning that patients with HF would be at a high risk of financial catastrophe.

In our study, we sought to further develop the understanding of the causes and cost of HF treatment in a low-middle-income economy by describing the primary causes at Moi Teaching and Referral Hospital (MTRH), Kenya, and determining the direct medical cost of hospitalization among patients admitted with acute decompensated heart failure (ADHF).

## Methodology

### Study design and setting

This was a prospective study conducted at the Moi Teaching and Referral Hospital (MTRH), Kenya, over a period of three months, from 1 September to 30 November 2022. MTRH is a national referral hospital located in Western Kenya, with a catchment area of about 24 million people. It has two wings: a public wing and a private wing, with the former being where the general public seeks treatment. All those who were hospitalized in the public wing medical wards and cardiac care unit (CCU) with the diagnosis of ADHF based on the Modified Framingham clinical criteria for HF and were aged 18 years and above were included in the study. Those who were less than 18 years old, readmitted during the study period, and discharged to or from the MTRH private wing were excluded from the study.

### Case definitions

Comprehensive clinical data, including clinical symptoms and signs, past medical history, and results of laboratory as well as imaging investigations, were gathered and collated with the two-dimensional Doppler echocardiography (ECHO) and 12-lead electrocardiography (ECG) findings. Both electrocardiograms and echocardiograms were performed by well-trained ECHO/ECG technicians at the MTRH ECG/ECHO centre, according to the American Society of Cardiology guidelines (13). The findings were summarized by the principal investigator and further confirmed by a consultant cardiologist. The most likely primary cause of HF was then established using a predetermined case definition criteria (Supplemental Table 1), which was guided by the European Society of Cardiology guidelines for HF (14). Notably, similar criteria were previously applied in the Heart of Soweto study in South Africa (2,15), and the Sub-Saharan Africa Survey of Heart Failure (THESUS-HF study), which recruited participants from 9 African countries, Kenya included (4). The criteria were, however, tweaked to fit our setup with limited diagnostic resources.

**Figure 1:**
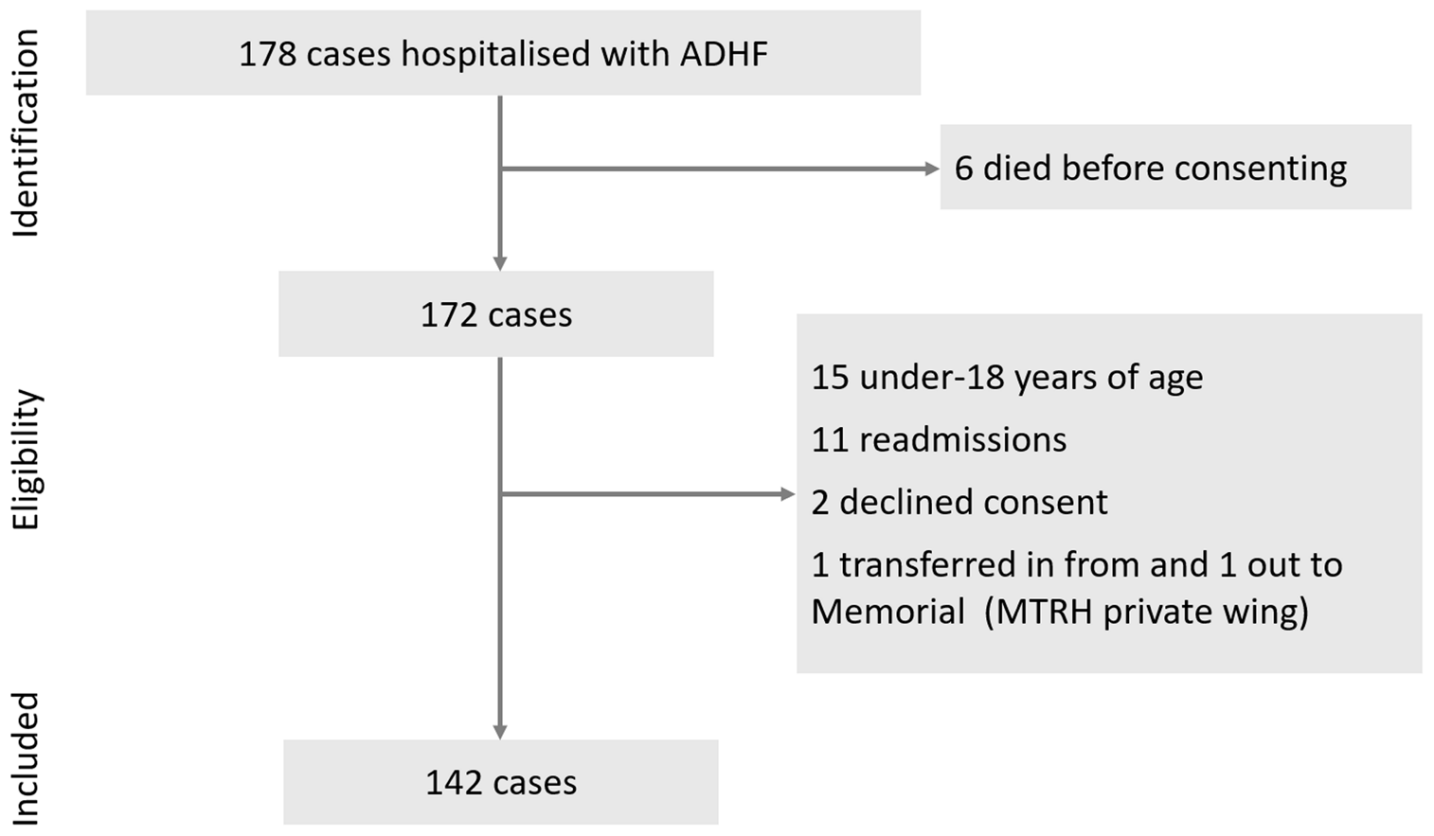
Recruitment schema of the participants. Note: 178 patients were clinically diagnosed to have acute decompensated heart failure. Those who were readmitted during the study period were excluded from the study to avoid recounting. Since the cost of treatment significantly varied between the public wing and the private wing of MTRH, the two patients who received care through the private wing were excluded. ADHF, acute decompensated heart failure; MTRH, Moi Teaching and Referral Hospital.

### Direct medical costing approaches

Costing was done using payers’ perspective (16), with a prospective prevalence-based method (17). Detailed data regarding direct medical costs were obtained from the MTRH finance department in printed forms. The data included a breakdown of all the costs that each patient incurred during hospitalization, from the date of admission to the point of discharge (the date on which the attending physician decided that the patient be discharged or the patient died). Micro-costing technique was used to detail the cost components, which included costs related to inpatient charges (nutrition, admission, physiotherapy, bed and nursing charges), medications, laboratory investigations, imaging, oxygen therapy, and other utilities (consumables such as syringes, gauzes, needles, nasal prongs, among others) (18,19). Further, direct costs included both out-of-pocket (OOP) and national health insurance (NHIF) payments. Notably, being a public health facility with human resource remuneration largely covered by the government, charges related to remuneration were not included. Moreover, indirect costs such as transport and productivity losses were not included.

### Data collection

Data were collected using interviewer-administered questionnaires and included information on sociodemographic characteristics, past medical history, New York Heart Association assessment (NYHA), vital signs such as blood pressure and heart rate, ancillary laboratory investigations such as haemoglobin, electrolytes, urea and creatinine, lipid profile, thyroid function tests, liver function tests, and international normalizing ratio, chest X-ray, select ECG, and ECHO findings. Comorbidities were physician-diagnosed and documented in the files, largely based on the patients’ past medical history and laboratory investigations.

### Statistical analysis

Data were analysed using RStudio statistical software version R.4.2.2. Continuous variables were reported using mean and median with respective standard deviations (SD) and interquartile range (IQR). Primary causes of HF were reported using proportions and percentages, with respective 95% confidence intervals (CI). Costs were reported using mean (SD) as cost (in Kenyan shillings, KES) per patient per day, calculated by dividing the total cost during hospitalization by the mean length of hospital stay (LOS) by the total number of participants, N, and compared between the CCU and the general ward. A linear regression model with standardized β-coefficients was used to determine how each cost component drove the overall cost. Due to the inability to delineate the direct medical cost of comorbidities, the mean costs were all-inclusive. However, the effect of each comorbidity on the overall cost was explored using both bivariate and multivariate generalized linear regression models with gamma distribution and log-link function. Comparisons of mean costs between the general ward and CCU were done using Mann-Whitney and Kruskal-Wallis non-parametric tests of association. Further, sub-analysis of the overall costs was done by NYHA, grade of ejection fraction, and in-hospital outcome (discharged alive or dead). Costs were further converted to American dollars (USD) per the Central Bank of Kenya foreign exchange rates during the study period, for which 1 USD = KES 122.6912 (20). Besides, since the study was conducted for a period shorter than 1 year, the costs were neither discounted nor adjusted for inflation.

### Ethical approval

This study was approved by the Moi University Institutional Research Ethics Committee (approval number 003738 and reference number IREC/2020/187). Formal written and signed informed consent was obtained from the participants before enrollment into this study. The informed consent described the details of the study, detailing both the benefits and potential harms. Participants’ data were decoded anonymously for confidentiality.

## Results

### Baseline characteristics of the study participants

A total of 142 participants were included in the study after 178 cases were screened for eligibility (Figure 1). The mean age was 54 (SD 20) years, with the median age of 60 (IQR 37.3, 70). 51.4% were females. Further, 73.9% had unskilled labour, and only 52.8% had NHIF cover. The mean length of hospital stay was 10.1 (SD 7.1) days. Table 1 shows further socio-demographic, laboratory, comorbidy, medication, and ECG/ECHO findings.

**Table 1:** Baseline characteristics of the participants.


BASELINE CHARACTERISTICS		(n, %)

**Gender**	Male	69 (48.59)

	Female	73 (51.41)

**Age (years) (mean (SD) | Median (IQR)** ^1^		54.0 (20.0) | 60 (37.3, 70)

**Marital Status**	Married	92 (64.8)

	Single	25 (17.6)

	Divorced/Separated	7 (4.93)

	Widowed	18 (12.7)

**Level of Education**	Primary	85 (59.9)

	Secondary	36 (25.4)

	Tertiary	21 (14.8)

**Occupation**	Unskilled	105 (73.9)

	Students	11 (7.7)

	Skilled	15 (10.6)

	Professional	8 (5.6)

	Retired	3 (2.1)

**Mode of Payment**	NHIF^2^	75 (52.8)

	OOP	67 (47.2)

**LOS (mean (SD)| median (IQR))** ^1^		10.1 (7.1) | 8 (5, 12.8)

**Ward**	General ward	73 (51.4)

	CCU	69 (48.6)

**Discharge Status**	Alive	115 (81.0)

	Dead	27 (19.0)

**HF History**	De novo HF	68 (47.9)

	ADCHF	74 (52.11)

**Comorbidities**	HIV	8 (5.6)

	HTN	45 (31.7)

	DM	17 (12.0)

	Anaemia	37 (26.1)

	Chronic Lung Disease^3^	41 (28.9)

	Liver Disease	47 (33.1)

	Renal Insufficiency	97 (68.3)

	Thyroid Dysfunction	26 (18.3)

	Dyslipidaemia	87 (61.3)

	Cancers	6 (4.2)

**NYHA Stage**	II	2 (1.4)

	III	87 (61.2)

	IV	53 (37.3)

**ECG/ECHO summary**	HR (bpm)^1,4^	105 (29) | 102 (85, 121)

	Sinus	110 (77.5)

	Atrial fibrillation	32 (22.5)

	Reduced	70 (49.3)

	Mildly reduced	18 (12.7)

	Preserved	54 (38.0)

	RVSP^1^	60 (20) | 56 (45, 75)

	TAPSE^1^	1.5 (0.5) | 1.3 (1, 1.9)

**Medications** ^5^	Diuretics	131 (92.3)

	MRAs	83 (58.5)

	ACEIs/ARBs	74 (52.1)

	SGLT2 Inhibitors	35 (24.7)

	Vasopressors	32 (22.5)

	B-Blockers	30 (21.1)

	Digoxin	29 (20.4)

	Amiodarone	27 (19.0)

	CCBs	19 (13.4)


Note: ^1^Values are both mean (SD) and median (IQR), respectively. ^2^NHIF was the main public health insurance cover in Kenya at the time of this study. ^3^Chronic lung diseases (CLDs) included chronic obstructive lung disease (19, 46.3% of the CLDs), pulmonary hypertension excluding pulmonary embolism (10, 24.4%), post-TB lung disease and bronchiectasis (7, 17.1%), lung masses (3, 7.3%), and pulmonary embolism and chronic thromboembolic pulmonary hypertension (2, 4.9%). ^4^Heart rate was measured during admission. ^5^The list of the medications that the patients received during admission, and the list is not mutually exclusive. ACEIs, Angiotensin-converting enzyme inhibitors; ADCHF, acute decompensation of chronic heart failure; ARBs, Angiotensin receptor blockers; CCB, calcium channel blockers; CCU, cardiac care unit; DM, diabetes mellitus; HIV, human immunodeficiency virus; HR, heart rate; IQR, interquartile range; MRAs, mineralocorticoids; NHIF, National Health Insurance Fund; OOP, out-of-pocket payment; RVSP, right ventricular systolic pressure; SD, standard deviation; SGLT2, sodium glucose transporter-2; TAPSE, transannular plane systolic excursion.

### Primary causes of HF

**Figure 2:**
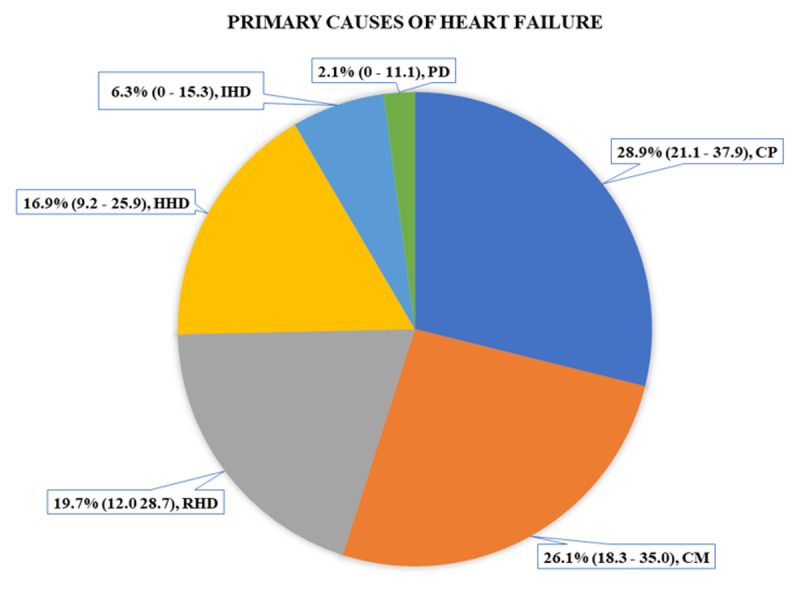
Primary causes of heart failure. Note: The values are presented as percentages, with 95% confidence intervals in round brackets. CM, cardiomyopathy; CP, cor pulmonale; HHD, hypertensive heart disease; IHD, ischemic heart disease; PD, pericardial heart disease; RHD, rheumatic heart disease.

The most common primary cause of HF was cor pulmonale (CP), accounting for 28.9% (95% CI 21.1%–37.9%) of the cases, followed by dilated cardiomyopathy (CM), 26.1% (95% CI 18.3%–35.0%), rheumatic heart disease (RHD), 19.7% (95% CI 12%–28.7%), and hypertensive heart disease (HHD), 16.9% (9.2%–25.9%) (Figure 2).

### Direct medical cost of HF hospitalization

**Figure 3:**
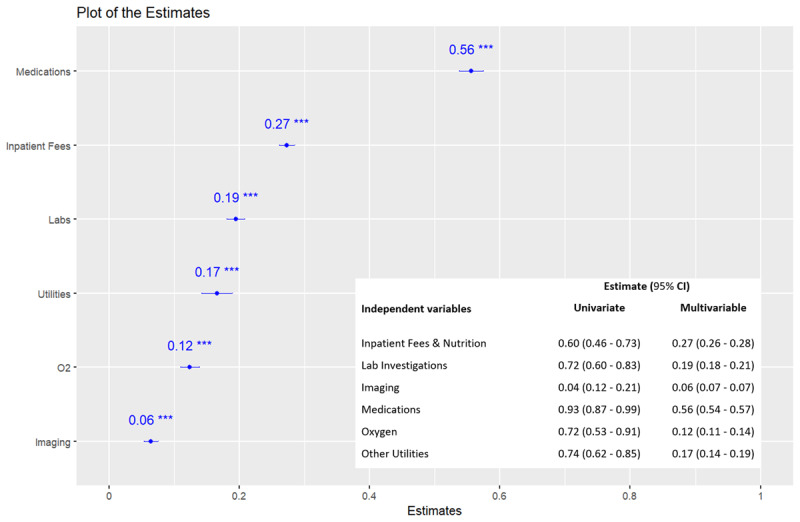
Drivers of the overall direct medical cost of HF hospitalization. Note: The β-regression coefficients were derived using generalized linear models with gamma distribution and log link function and reflect the unit change in the overall cost per unit change in the cost of medications, inpatient fees, labs (laboratory investigations), utilities, O_2_ (oxygen therapy), and imaging. The units were standardized for the purpose of ranking. CI, confidence interval.

**Figure 4:**
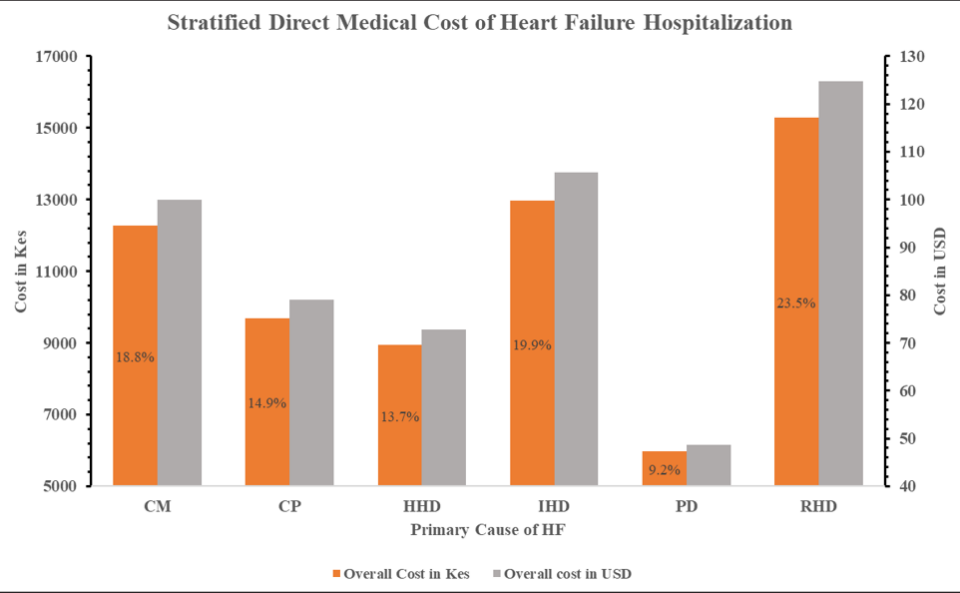
Stratified direct medical costs by the primary cause of HF. Note: The figure shows the overall direct medical cost of heart failure hospitalization per patient per day, presented in Kenyan shillings (KES) on the left vertical axis and US dollars (USD) on the right vertical axis for ease of international comparison. The percentages in the bars represent the proportion of the overall cost incurred by each primary cause of heart failure. CM, cardiomyopathy; CP, cor pulmonale; HHD, hypertensive heart disease; PD, pericardial disease; RHD, rheumatic heart disease.

The mean direct medical cost of HF hospitalization was KES 11,470.94 (SD 8,289.57) [USD 93.49 (67.56)] per patient per day, with the cost in the CCU being as twice as much in the general ward (Table 2). Of the cost components, the cost of medications was the leading driver of the overall cost, with a standardized β-coefficient of 0.56 (95% CI 0.55–0.56). This meant that an increase in the cost of medication by one unit led to an increase in the overall cost by 0.56 standard deviation units (Figure 3). Absolutely, the cost of medications was KES 36,694.92 (SD 42,403.40) [USD 299.08 (345.61)] per patient during the hospitalization period, comprising 35.2% of the overall costs (Table 2, Supplementary File 1, Figure 1). Stratified by the primary cause of HF, RHD incurred the highest mean direct medical cost, KES 15,299.08 (SD 13,196.89) [USD 124.70 (107.56)] per patient per day, accounting for 23.5% of the overall costs (Figure 4) and costing twice as much in the CCU as in the general ward (Table 3). Overall, the direct medical cost of HF hospitalization was increasingly higher among those with NYHA stages III and IV, HFrEF and HFmrEF, comorbid renal and liver diseases, and those who died during hospitalization (Supplementary File 1, Supplementary Tables 1, 2, 3, and 4; Supplementary File 1, Supplementary Figure 1).

**Table 2:** Direct medical cost of heart failure hospitalization.


ITEM LINE		OVERALL COST	% OF OVERALL	OVERALL COST BY WARD	

				GENERAL WARD	CCU

Inpatient Fees	KES USD	18091.72 (20861.32) [147.46 (170.03)]	17.4	7919.78 (4856.96) [64.55 (39.59)]	28853.34 (25471.93) [235.17 (207.61)]

Lab Investigations	KES USD	22733.39 (14135.5) [185.29 (115.21)]	21.8	20953.01 (11167.76) [170.78 (91.02)]	24619.99 (16590.92) [200.67 (135.23)]

Imaging	KES USD	5139.44 (4928.71) [41.89 (40.17)]	4.9	6434.25 (5814.52) [52.44 (47.39)]	3769.57 (3296.85) [30.72 (26.87)]

Medications	KES USD	36694.92 (42403.40) [299.08 (345.61)]	35.2	18833.61 (19719.78) [153.50 (160.73)]	55591.67 (51105.17) [453.10 (416.53)]

Oxygen	KES USD	10044.44 (9743.60) [81.87 (79.42)]	9.6	7960.53 (6530.91) [64.88 (53.23)]	12373.53 (12076.73) [100.85 (98.43)]

Other Utilities	KES USD	11427.48 (12925.69) [93.14 (105.35)]	11.0	7505.31 (4583.15) [61.17 (37.36)]	15577.02 (17035.38) [126.96 (138.85)]

**Total cost/patient (KES)**		**99065.65 (76339.71)**		**65775.94 (36922.10)**	**134285.20 (90510.95)**

**Total cost/patient [USD]**		**[807.44 (622.21)]**		**[536.11 (300.94)]**	**[1094.50 (737.71)]**

**LOS (days)**		**10.1 (7.1)**		**10.0 (6.6)**	**10.3 (7.7)**

**Cost/patient/day (KES)**		**11470.94 (8289.57)**		**7687.57 (3783.92)**	**15473.63 (9782.79)**

**Cost/patient/day [USD]**		**[93.49 (67.56)]**		**[62.66 (30.84)]**	**[126.12 (79.74)]**


Note: The values are in Kenyan Shillings (KES) but also converted to United States of America Dollars (USD) in the square brackets. LOS, length of hospital stay; SD, standard deviation.

**Table 3:** Direct medical cost per patient per day per primary cause of HF.


PRIMARY CAUSE OF HF	OVERALL COST	OVERALL COST: GENERAL WARD	OVERALL COST: CCU

CM	12268.08 (7816.12) [99.99 (63.71)]	7145.15 (2825.26) [58.24 (23.03)]	16171.26 (8184.52) [131.80 (66.71)]

CP	9696.38 (5646.20) [79.03 (46.02)]	7471.55 (3292.20) [60.90 (26.83)]	14488.33 (6741.84) [118.09 (54.95)]

HHD	8934.33 (3757.89) [72.82 (30.63)]	8719.35 (3914.86) [71.07 (31.91)]	9579.26 (3490.76) [78.08 (28.45)]

IHD	12966.47 (6656.49) [105.68 54.25)]	0.00	12966.47 (6656.49) [105.68 (54.25)]

PD	5968.67 (1542.23) [48.65 (12.57)]	6166.27 (2126.65) [50.26 (17.33)]	5573.44 [45.43 (45.43)]

RHD	15299.08 (13196.88) [124.70 (107.56)]	7598.44 (6359.84) [61.93 (51.84)]	18946.76 (14126.75) [154.43 (115.14)]


Note: The costs are presented as mean and corresponding standard deviation in round brackets. They are also stratified as overall costs (both general ward and CCU) and by ward (general ward vs. CCU). The units are in Kenyan shillings (KES) but also converted to US dollars (USD) in the square brackets. CCU, cardiac care unit; CM, cardiomyopathy; CP, cor pulmonale; HHD, hypertensive heart disease; IHD, ischemic heart disease; PD, pericardial disease; RHD, rheumatic heart disease.

## Discussion

Our study adds significant value to the knowledge of the epidemiological profile of acute heart failure and its direct medical costs of treatment during hospitalization in Kenya, where such information is still scarce in the face of the ongoing epidemiological transition.

### Primary causes of heart failure

We found that CP was the leading cause of HF, largely contrasting findings from prior studies which reported that HHD, DCM, and RHD were the leading primary causes of HF in Sub-Saharan Africa (21,22,23,24,25). It is possible that the use of different study designs and the lack of consistent criteria for determining primary HF aetiologies resulted in the widely varying proportions in Table 4. More than three-quarters of the patients in the studies by Kwan et al. (25) and Karaye et al. (26) were outpatient, and it is not clear if the primary HF aetiological profile in the outpatient setup would be different from that of hospitalized patients. Furthermore, limited diagnostic capacity in the different regions in SSA could influence determination of primary HF causes (27,28). Notwithstanding, the predominance of CP in our study, being only comparable to The Heart of Soweto Study which found 27% in South Africa (2,15), was striking, hence the need for further exploratory studies. Our study further confirmed the predominance of non-ischemic causes of HF in SSA (29,30,31).

**Table 4:** Primary causes of HF of in Sub-Saharan Africa: Comparison of the current study and prior studies.


AUTHOR	N	STUDY DESIGN	Country	HHD	CM	RHD	CP	IHD	PD

Amoah & Kallen, 2000	572	Prospective	Ghana	21.3	16.8	20.1		9.8	7.7

Ismail et al., 2007	65	Prospective	Uganda		47.7	35.4	4.6	4.6	1.5

Sliwa et al., 2008	4162	Prospective	South Africa	35.0	28.0	8.0	27.0	9.0	

Damasceno et al., 2012	1009	Prospective, multicentre	9 African countries	45.4	18.8	14.3		7.7	6.8

Owusu & Adu-Boakye, 2013	398	Cross-sectional	Ghana	45.0	15.0	23.0	1.2	2.3	2.2

Kwan et al., 2013	138	Prospective	Rwanda	8.0	54.0	25.0			

Ogah et al., 2014	452	Prospective	Nigeria	78.5	7.5	2.4	4.4	0.4	3.3

Abebe et al., 2016	311	Retrospective	Ethiopia	16.0	12.5	40.1	4.5	15.8	

Makubi et al., 2016	411	Prospective, matched	Tanzania & Sweden	47.0	23.0		3.0	10.0	

Kingery et al., 2017	145	Prospective	Tanzania	42.8	19.3	16.6	7.6	6.2	

Eberly et al., 2018	451	Prospective	Rwanda	13.1	39.7	26.8			

Karaye et al., 2021	1294	Prospective, multicentre	5 African countries	35.0	15.9	7.2		20.0	

Oyoo & Ogola, 1999	91	Cross-sectional	Kenya, KNH	17.6	25.2	32.0		2.2	13.2

Ogeng’o et al., 2014	116	Prospective	Kenya, KNH	15.5	18.1	12.9		9.5	

**Current study**	**142**	**Prospective**	**Kenya, MTRH**	**16.9** 9.2–25.9	**26.1** 18.3–35	**19.7** 12–28.7	**28.9** 21.1–37.7	**6.3** 0–15.3	**2.1** 0–11.1


Note: CM, cardiomyopathy; CP, cor pulmonale; HHD, hypertensive heart disease; IHD, ischemic heart disease; KNH, Kenyatta National Hospital; MTRH, Moi Teaching and Referral Hospital; PD, pericardial disease.

The current study highlights the changing landscape of primary causes of HF in SSA, particularly of CP and chronic lung diseases, alongside other major causes of HF (32,33,34). Notably, 46.3% of those who had CP in this study had chronic obstructive lung diseases, 24.4% had indeterminate pulmonary hypertension, 17.1% had post-tuberculosis lung fibrosis and bronchiectasis, 7.3% had lung masses, and 4.9% had pulmonary embolism. Although we did not collect data on indoor air pollution or interrogate causal pathways to CP, it is possible that indoor air pollution contributed to the high prevalence of chronic lung diseases, hence CP. Recent studies conducted in Western Kenya reported significantly high levels of indoor air pollution due to biomass fuel use (35,36), and this was strongly associated with different cardiac abnormalities, differentially affecting women more than men and those with low socioeconomic status (37,38). It is notable that more women than men had HF and CP in our study; 51.4% vs. 48.6%, and, 56.1% vs. 43.9% respectively. Additionally, 73.9% of the participants had unskilled labour, reflecting low socioeconomic status. Further studies are therefore needed to explore CP as an additional important emerging cause of HF in SSA and to elucidate causal relationships in both inpatient and outpatient settings, including important social factors such as indoor air pollution, particularly with the current changing landscape and factors of public and global health concern such as climate change.

### Direct medical cost of heart failure hospitalization

This study found that the mean direct medical cost of HF hospitalization at MTRH was KES 11,470.94 (SD 8,289.57) [USD 93.49 (67.56)] per patient per day. A study in Nigeria found a cost of USD 2,128 per patient per year (8), while a study in Ghana reported a median direct medical cost of USD 182 per hospitalization (39). Evidently, the reported costs are quite varied, attributable to differences in the costing methodologies (Table 5). For instance, Ogah et al. used a societal perspective (8), while Appiah et al. did not include out-of-pocket payments (39).

**Table 5:** Comparison of cost of HF treatment in different countries by GDP-PPP.


A	B	C	D	E	F	G	H	I

STUDY	COUNTRY	YEAR OF STUDY	COST BY YEAR OF STUDY (USD)	GDP-PPP BY YEAR OF STUDY	STANDARDIZED COST PER GDP-PPP	MEASURE	COST AS	PERSPECTIVE

**Current study**	Kenya	2023	93.49	6,307.20	0.01	Mean	Per patient per day	Payer’s

**Current study**	Kenya	2023	807.44	6,307.20	0.13	Mean	Per patient per hospitalization	Payer’s

Appiah et al.	Ghana	2023	182	7,543.00	0.02	Median	Per hospitalization	Payer’s

Ong et al.	Malaysia	2022	1473	34,366.20	0.04	Mean	Per patient per year	Payer’s

Alghamdi et al.	Saudi Arabia	2021	9563	55,768.20	0.17	Mean	Per patient per year	Payer’s

Ogah et al.	Nigeria	2014	2128	5,252.20	0.41	Mean	Per patient per year	Societal

Zaour et al.	Canada	2015	10123	44,669.50	0.23	Mean	Per patient per hospitalization	Payer’s

Kwok et al.	US	2021	11845	71,318.30	0.17	Mean	Per patient per hospitalization	Payer’s


Note: GDP-PPP, gross domestic product by purchasing power parity; USD, United States dollars. GDP-PPP refers to the GDP of a given country converted into international dollars using the country’s relative purchasing power parity (40). It considers varying pricing levels and economies in different countries. GDP-PPP by year of study represents the GDP-PPP of the country in the year in which the study was published. Standardized cost per GDP-PPP (column F) was derived by dividing the cost of heart failure (column D) by the GDP-PPP of the respective country by the year of the study (column E), with the lower the value, the cheaper the cost relative to the country’s economy.

Besides, it should be noted that the cost of treatment of HF varies significantly by different economies. In the high-income countries, Kwok et al. found USD 11,845 (SD 22,710) in the United States (41), while Zaour et al. found CAD 10,123 per patient per hospitalization in Canada (42). In Asia, the cost of HF was USD 9,563 per patient per year in Saudi Arabia (43) and USD 1,473 per patient per year in Malaysia (44). Standardizing the costs by the country-specific gross domestic product per purchasing power parity (GDP-PPP) (40), however, revealed that our finding was relatively cheaper compared to other economies (Table 5). Expectedly, different healthcare resource utilization alongside different times, costs of health system inputs and markets, and heterogeneity in the study designs explain the differences observed (45,46,47,48). On the other hand, our cost estimates represent charges in a government-subsidized health care system wherein indirect costs as well as the human resource costs are not charged directly to patients, hence the lower costs. Importantly, these findings highlight the need to interpret the cost of HF treatment in the context of the study.

The 2022 Economic Survey by the Kenya National Bureau of Statistics indicated that the gross national income per capita in 2021 was KES 20,122.23 (USD 164.01) per month (49). Even though we were not able to collect data on the average monthly income of the participants in this study owing to the large proportion of casual workers, it is notable that 83.5% of the participants did not complete high school and 73.9% were unskilled labourers, reflecting low socioeconomic status. Therefore, contextually, the cost of treatment for HF was extremely expensive against the Kenyan gross national income per capita, posing a substantial risk of financial catastrophe to the affected families and public healthcare system. It is further notable that just about half of the participants had national health insurance coverage in our study, with the rest being out-of-pocket payments. Even though this was above the Kenyan national health insurance coverage rate of 26.5% at the time of the study (9), there is still a dire need to intensify advocacy for and implementation of widespread and comprehensive health insurance coverage among patients with HF towards the achievement of universal health coverage (UHC) and sustainable development goals (50,51). Further, there is a need to intensify primary management and prevention strategies among patients with HF to reduce chances of hospitalization, hence the high direct medical costs of HF treatment.

To the best of our knowledge, this was the first study in the region to stratify the direct medical cost of HF hospitalization based on the primary causes. We found that RHD was the most expensive primary cause of HF to treat, incurring KES 15,299.08 (13,196.88) [USD 124.70 (107.56)] per patient per day. Sub-analysis showed that of the patients with RHD, 67.9% were admitted to the cardiac intensive care unit (CCU). Further, RHD-associated HF accounted for 27.5% and 28.6% of the total CCU HF admissions and deaths respectively, comprising 29.6% of all those who died in this study (Supplementary File 1, Supplementary Table 5). Noting that the direct medical cost of those who died was twice the cost of those who were discharged alive, it is possible that the high cost of management of RHD was driven by the adverse outcome of in-hospital mortality, hence the need for early detection and treatment. Furthermore, RHD also accounted for 37.8% of all the patients with atrial fibrillation in this study; hence, additional treatment modalities such as cardioversion and anticoagulation among patients with RHD and atrial fibrillation (52) increased the cost of treatment. Additional studies are therefore recommended to extensively characterize costs attributable to RHD. Moreover, noting that RHD, among other leading causes of HF, are completely preventable, primary care and community-based prevention measures, including eradication strategies, need to be implemented to mitigate HF hospitalization and hence adverse outcomes and cost of treatment.

Reflectively, CP, despite being the most common primary cause of HF in this study, was the fourth most expensive to treat, costing KES 9,696.38 (5,646.20) [USD 79.03 (46.02)] per patient per day. This is because CP accounted for most HF admissions in the general ward and HF cases discharged alive: 38.4% and 30.4%, respectively (Supplementary File 1, Supplementary Table 5). Notably, the overall cost of treatment among those hospitalized in the general ward and among those discharged alive was less than half that of those hospitalized in the CCU or who died, as also evidenced in prior studies (42,53,54). The economic impacts of CP on households and potential for causing financial catastrophe cannot however be overlooked.

The cost of medications was the leading driver of the overall cost in this study, absolutely comprising 35.2% of the overall costs. This contrasts the findings by Ogah et al. (8), Ong et al. (44), and Alghamdi et al. (43), who reported that the cost of hospitalization, including procedures such as implantable cardioverter defibrillators (ICD), percutaneous coronary intervention (PCI), and cardiac resynchronization therapy (CRT), was the leading driver, accounting for 30.5–71% of the overall costs, with the cost of medication contributing only 11–15%. Conversely, our study did not include the costs of such procedures since they were not readily available. Furthermore, our study described costs among hospitalized patients only, while the prior studies reported both inpatient and outpatient costs, which could lead to different findings. Arguably, the high proportion of the cost of medications in our study was driven by intensive care in the CCU, including the cost of additional expensive medications such as vasopressors. Notably, the cost of medications in the CCU was about three times the cost in the general ward, and about 22% of the participants required vasopressors. It is notable that the wide variation of institution-specific treatment protocols and medication prices across different countries would contribute to different results, further explaining the relatively lower cost of medications observed in our study. Moreover, the prices of medications are much higher in the high-income vs. low- and middle-income countries (55,56).

We found that the direct medical cost of HF hospitalization increased with worsening NYHA functional status, consistent with the findings in Malaysia (44), Saudi Arabia (43), Greece (57), Poland (54), and Spain (58). Further, those with reduced and mildly reduced ejection fractions also incurred higher costs than those with preserved ejection fraction, which is consistent with previously published literature (57,59,60). This reflects high resource utilization among hospitalized patients with advanced HF, reinforcing the need for early optimization of HF treatment to prevent progression. Moreover, this study indicated that the presence of comorbidities such as liver disease and renal impairment increased the overall direct cost of HF hospitalization with statistical significance on a multivariable regression model, consistent with prior studies (43,57,60,61). Patients with HF have been shown to have multiple comorbidities, which significantly influence the direct medical cost of hospitalization by increasing the length of hospital stay and hence resource mobilization, ultimately translating into higher costs (62,63,64). Renal impairment, particularly, is a common finding among patients with HF, and up to 30% of patients with HF could experience worsening of renal function, thus influencing HF hospitalization costs and outcomes (65). In our study, the presence of renal impairment increased the overall cost by 35%. On the other hand, the finding that the presence of liver disease significantly increased the overall cost of HF in this study was new, having not been explored in prior studies. This calls for further cost-of-illness studies to explore the absolute economic contribution of liver disease among other comorbidities in the management of HF. Our findings further emphasize the need for early detection and management of comorbidities among patients with HF to mitigate the cost of treatment and hence the economic burden on the patients and healthcare system.

Epidemiologically, it should be emphasized that HF affects a relatively younger population in SSA than in the high-income countries (66,67). Our study, consistent with prior literature, found a mean age of 54 years, with a slight female predominance, 51.4% (68). Acknowledging the pivotal role of the young population in forming the workforce and driving the economy in SSA, the extremely expensive direct medical cost of HF hospitalization, as found in this study, could threaten this population into financial catastrophe and limit their ability to recover and operate optimally.

### Limitations

This study had a number of limitations. First, advanced cardiac diagnostic modalities such as cardiac computed tomography (CT), cardiac magnetic resonance imaging, coronary CT angiography, or cardiac catheterisation, which are key in evaluating aetiologies of HF, were not available; hence, misclassification of some of the primary causes of HF was inevitably possible. Second, the relatively smaller sample size of the study and recruitment of participants from one public national referral hospital where healthcare services are subsidized could limit generalizability and application of the findings to private healthcare facilities. Third, the study was conducted over a period shorter than a year; hence, the costs were not discounted or adjusted for inflation. Consequently, it may be difficult to extrapolate the findings over a longer time horizon, hence the need for more robust time-series analysis to aid in long-term prediction of the cost of management of HF. However, the costs reported per patient per day are intuitive for patient education and empowerment as they reflect daily consumption of healthcare resources during hospitalization. Fourth, the cost of procedures such as PCI, CRT, ICD, and valvular repairs was not included. Therefore, the values in this study may be significantly underestimated. The investigators, however, had no control over the procedures due to their lack of availability. Finally, outpatient as well as indirect costs such as transportation and loss of productivity from work were not included; hence, the exact value of the economic burden of HF management cannot be inferred. Recognizing the mentioned limitations, this study adds valuable insight to the existing literature on the causes of HF in SSA in view of the changing landscape. To the best of our knowledge, this study is the first to report the cost of treatment of HF stratified by primary causes. It therefore provides important data pivotal in improving HF patient-centred care and guiding healthcare resource allocation.

## Conclusion

In conclusion, cor pulmonale was the most common primary cause of HF, followed by CM, RHD, and HHD. The mean direct medical cost of hospitalization was KES 11,470.94 [USD 93.49] per patient per day. Weighted against Kenyan monthly gross national income per capita, this was extremely expensive. Further stratified by primary cause of HF, RHD incurred the highest direct medical costs. To the best of our knowledge, this was the first study in the region to stratify the direct medical cost of HF hospitalization by the primary causes. Therefore, policies and community-oriented interventions should be put in place for early recognition and treatment of CP, among other primary causes of HF, in view of the ongoing epidemiological transition in SSA, since these causes are completely preventable. Further studies are also recommended to explore the causal relationship between wider causes of HF in SSA, particularly with the current public health challenges of global concern such as climate change and air pollution. Additionally, widespread and improved health insurance coverage should be implemented to protect the affected families and the healthcare system from catastrophic spending resulting from the high direct medical cost of HF hospitalization amid the efforts towards the achievement of universal health coverage in Kenya. Finally, more extensive cost-of-illness studies, such as societal perspective, are recommended, detailing lost productivity among patients hospitalized with HF.

## Data Accessibility Statement

Data used in this study is available for sharing upon reasonable request to the corresponding author.

## Additional File

The additional file for this article can be found as follows:

10.5334/gh.1426.s1 Supplementary File 1. Supplementary Tables 1 to 6 and Supplementary Figures 1 to 2.
