# Supplementary material for: Primary Causes and Direct Medical Cost of Heart Failure Among Adults Admitted with Acute Decompensated Heart Failure in a Public Tertiary Hospital, Kenya
Source: Glob Heart. 2025 May 2;20(1):42. doi: 10.5334/gh.1426 (PMC12047636; doi:10.5334/gh.1426)
Supplement: Supplementary File 1. — Supplementary Tables 1 to 6 and Supplementary Figures 1 to 2. [file gh-20-1-1426-s1.pdf]

Supplementary Files

Supplemental material

Article Title: Primary causes and direct medical cost of heart failure hospitalization in a public tertiary hospital, Kenya

Authors: Victor M. Wauye, Chrispine O. Oduor, Felix A. Barasa, G. Titus Ngeno

Cause of HF	Case Definition
Hypertensive Heart Disease	Documented history or new diagnosis of HTN plus ECHO report of LVH or ECG features of LVH: LAD or Positive Cornell Voltage ECG criteria for LVH (Sum of R wave in aVL and S wave in V3 > to 2.8mV in males and 2.0mV in females) or Sokolow-Lyon criteria: sum of S in V1 or V2 and R in V5 or V6 >35mm.
Rheumatic Heart Disease	Pathological MR with at least 2 of: AMVL thickening, chordal thickening, restricted leaflet motion or excessive leaflet tip motion during systole or<br>MS mean gradient ≥4mmHg or<br>Pathological AR with at least 2 of: AV focal thickening, coaptation defect, restricted leaflet motion or prolapse.
Ischemic Heart Disease	Regional wall abnormality plus/minus pathological Q waves defined as any Q waves >0.02s in V2-V3 or QS complex in V2-V3, or Q waves ≥0.03s and ≥1mm deep or QS complex in other leads in at least 2 contiguous leads.
Dilated Cardiomyopathy	Dilated ventricular chambers with reduced LVEF or dilated ventricular chambers with ECG features of LVH in the absence of HHD and IHD.
Cor Pulmonale	ECHO: Pulmonary HTN of >35mmHg (in the absence of valvular heart disease as RHD and LVEF ≤40%), TAPSE <1.7cm, right ventricular enlargement, and dilated non-collapsing IVC, and/or<br>ECG: Right Axis Deviation, R/S amplitude ratio>1 in V1, R/S amplitude ratio<1 in V6, $S_1Q_3T_3$, or Right Bundle Branch Block and/or<br>Documented chronic lung disease.
Pericardial Disease	Pericardial echo free fluid plus/minus any features of cardiac tamponade and indices of cardiac contusion in the absence of any identifiable causes above.

Supplemental Table 1: Case definition for the primary causes of heart failure

Note: The table shows how the most probable primary cause of heart failure was determined. Significant clinical history and physical examination findings were gathered and correlated with the characteristic ECG and ECHO findings. The findings were then discussed with the consultant cardiologist before arrival at the most probable primary cause of HF among the participants in this study**.** AR, Aortic valve; AMVL, Anterior mitral valve leaflet; AV, Aortic valve; ECG, Electrocardiogram; ECHO, Echocardiogram; HHD, Hypertensive heart disease; HTN, Hypertension; IHD, Ischaemic heart disease; IVC, Inferior vena cava; LAD, Left axis deviation; LVEF, Left ventricular ejection fraction; LVH, Left ventricular hypertrophy; MR, Mitral regurgitation; TAPSE, Transannular plane systolic excursion.

Item Line	II	III	IV
Inpatient Fees & Nutrition	22250.00 (20435.39) [181.35 (166.56)]	15866.59 (18860.78) [129.32 (153.73)]	21587.36 (23712.70) [175.95 (193.27)]
Lab Investigations	15000.00 (7636.76) [121.99 (62.24)]	21620.37 (13900.11) [176.22 (113.29)]	24833.40 (14579.20) [202.41 (118.83)]
Imaging	1700.00 [13.86]	5136.78 (5193.54) [41.87 (42.33)]	5273.59 (4559.27) [42.93 (37.16)]
Medications	13328.00 (9043.90) [108.63 (73.71)]	32281.26 (41244.36) [263.11 (336.16)]	44821.76 (44041.37) [365.32 (358.96)]
Oxygen	~ ~	9306.98 (9158.95) [75.86 (74.65)]	11137.93 (10622.23) [90.78 (86.58)]
Other Utilities	3043.40 (1266.29) [24.81 (10.32)]	9548.21 (8502.91) [77.82 (69.30)]	14828.69 (17692.37) [120.86 (144.20)]
**Cost/patient (Kes)**	**55821.40 (20294.53)**	**88944.94 (72939.86)**	**117310.76 (80080.19)**
**Cost/patient (USD)**	**454.97 (165.41)**	**724.95 (594.50)**	**956.14 (651.26)**
**Cost/patient/day (Kes)**	**7085.82 (4155.98)**	**9876.02 (6114.21)**	**14254.49 (10548.81)**
**Cost/patient/day (USD)**	**57.75 (33.87)**	**80.49 (49.83)**	**116.18 (85.98)**

Supplemental Table 2: Direct medical cost by NYHA

Cost presented in Kenya Shillings (Kes), and converted to US Dollars (USD) in the square brackets. NYHA, New York Heart Association.

Item Line	Reduced EF	Mildly Reduced EF	Preserved EF
Inpatient Fees & Nutrition	18517.05 (19336.09) [150.92 (157.60)]	28388.89 (30373.86) [231.38 (247.56)]	14107.97 (17963.52) [114.99 (146.41)]
Lab Investigations	24775.31 (14999.00) [201.93 (122.25)]	17556.67 (10023.06) [143.10 (81.69)]	21812.04 (13819.54) [177.78 (112.64)]
Imaging	18517.05 (19336.09) [150.92 (157.60)]	28388.89 (30373.86) [231.38 (247.56)]	14107.97 (17963.52) [114.99 (146.41)]
Medications	41591.83 (43608.97) [339.00 (355.44)]	32017.35 (42147.84) [260.96 (343.53)]	31906.28 (40931.24) [260.05 (333.61)]
Oxygen	10475.00 (8535.23) [85.38 (69.57)]	3400.00 (3645.09) [27.71 (29.71)]	10407.50 (10797.33) [84.83 (88,00)]
Other Utilities	13279.54 (16131.82) [108.24 (131.48)]	9987.20 (9204.80) [81.40 (75.02)]	9507.08 (8337.30) [77.49 (67.95)]
**Cost/patient (Kes)**	**106557.52 (78879.88)**	**93173.00 (70667.02)**	**91318.19 (75198.12)**
**Cost/patient (USD)**	**868.50 (642.91)**	**759.41 (575.97)**	**744.29 (612.91)**
**Cost/patient/day (Kes)**	**12463.27 (8072.83)**	**13418.63 (13936.74)**	**9535.35 (5409.38)**
**Cost/patient/day (USD)**	**101.58 (65.80)**	**109.37 (113.59)**	**77.72 (44.09)**

Supplemental Table 3: Direct medical cost by ejection fraction

Cost presented cost per patient per day in Kenya Shillings (Kes), and converted to US Dollars (USD) in the square brackets. EF, Ejection fraction.

	Estimate (CI, p-value)	
**Independent variables**	**Univariate**	**Multivariable**
HTN	0.82 (0.63 – 1.08, 0.159)	0.78 (0.59 – 1.02, 0.0728)
DM	0.86 (0.58 – 1.27, 0.457)	0.99 (0.67 – 1.47, 0.9647)
Chronic Lung Disease	0.78 (0.58 – 1.06, 0.12)	0.78 (0.57 – 1.06, 0.1169)
Cancer	0.82 (0.44 – 1.54, 0.542)	0.97 (0.53 – 1.76, 0.9150)
Liver Disease	**1.39 (1.06 – 1.81, 0.0181)**	**1.40 (1.08 – 1.81, 0.0120)**
Renal Impairment	**1.33 (1.03 – 1.73, 0.0308)**	**1.35 (1.04 – 1.77, 0.0279)**
Dyslipidaemia	0.98 (0.76 – 1.27, 0.883)	1.11 (0.87 – 1.41, 0.4191)
Thyroid Disease	1.22 (0.87 – 1.69, 0.246)	1.25 (0.92 – 1.70, 0.1621)
Anaemia	0.89 (0.67 – 1.20, 0.453)	0.97 (0.74 – 1.29, 0.8583)
HIV	0.64 (0.37 – 1.10, 0.111)	0.72 (0.42 – 1.23, 0.2291)
Atrial fibrillation	1.10 (0.81 – 1.49, 0.543)	0.99 (0.74 – 1.33, 0.9629)

Supplemental Table 4: Comorbidities that predicted the direct medical cost of heart failure hospitalization

CI, Confidence interval; DM, Diabetic mellitus; HIV, Human immunodeficiency virus; HTN, hypertension.

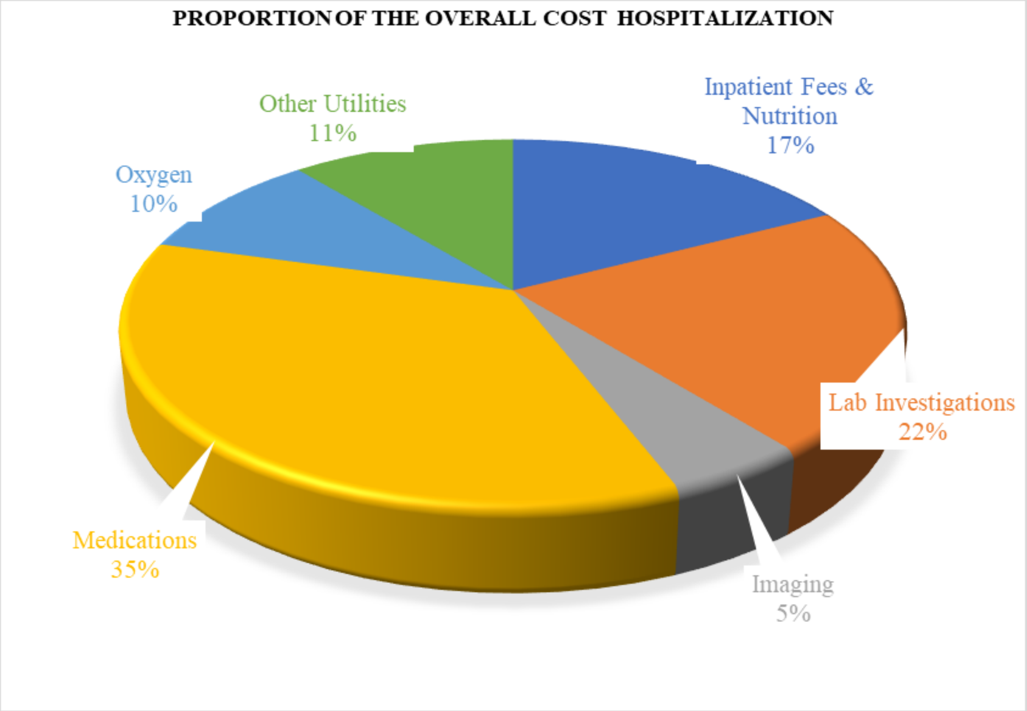

Supplemental Figure 1: Proportions of the cost components as a percentage of overall cost

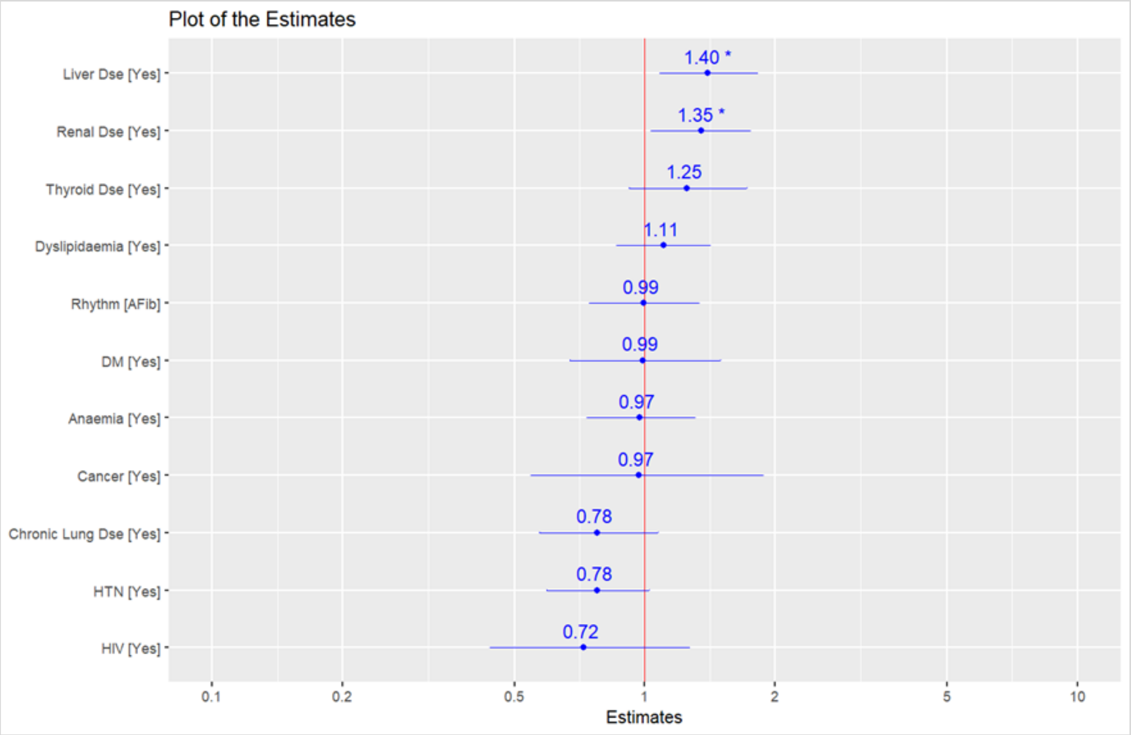

Supplemental Figure 2: Coefficient plot of the comorbidities that predicted the direct medical cost of HF hospitalization

DM, Diabetes mellitus; Dse, Disease; HTN, hypertension; HIV, Human immunodeficiency virus

**Item Line**	**Discharged alive**	**Died**	**P-value**
Inpatient Fees & Nutrition	18545.04 (21861.98) [151.15 (179.19)]	16160.88 (16115.31) [131.72 (131.35)]	0.835
Lab Investigations	21745.41 (13169.62) [177.24 (107.34)]	26941.48 (17332.89) [219.59 (141.27)]	0.190
Imaging	5482.61 (5300.07) [44.69 (43.20)]	3677.78 (2414.91) [29.98 (19.68)]	0.362
Medications	30007.95 (31990.84) [244.58 (260.74)]	65176.38 (64988.50) [531.22 (529.69)]	0.007
Oxygen	8667.31 (8330.68) [70.64 (67.90)]	13625.00 (12239.15) [111.05 (99.76)]	0.047
Other Utilities	10104.70 (12862.09) [82.36 (104.83)]	17061.51 (11831.63) [139.06 (96.43)]	<0.001
**Overall cost/patient (Kes)**	**89740.26 (65174.89)**	**138784.92 (104673.69)**	**0.014**
**Overall cost/patient (USD)**	**731.85 (531.51)**	**1131.81 (853.63)**	
**Overall cost/patient/day (Kes)**	**9642.05 (5389.56)**	**19260.63 (12941.75)**	
**Overall cost/patient/day (USD)**	**78.63 (43.95)**	**157.07 (105.54)**	

Supplemental Table 5: Direct medical cost by outcome

Cost presented in Kenya Shillings (Kes), and converted to US Dollars (USD) in the square brackets.

Item		CM	CP	HHD	IHD	PD	RHD
**Baseline Characteristics**							
Sex	Male (69)	23 (33.3)	18 (26.1)	11 (15.9)	6 (8.7)	2 (2.9)	9 (13.0)
	Female (73)	14 (19.2)	23 (31.5)	13 (17.8)	3 (4.1)	1 (1.4)	19 (26.0)
Age (years)	Mean (SD)	56.7 (18.6)	60 (18.4)	61.5 (16.9)	64.6 (9.9)	30 (13.1)	34.3 (14.6)
Duration of hospitalization (days)		11.3 (8.4)	10.5 (6.9)	10.6 (7.0)	6.7 (1.9)	11 (2.6)	8.8 (7.1)
		8 (6, 16)	8 (6, 13)	10 (5, 13.5)	7 (5, 7)	10 (9.5, 12)	6.5 (4, 11.3)
Ward	General Ward (73)	16 (21.9)	28 (38.4)	18 (24.7)	0	2 (2.7)	9 (12.3)
	CCU (69)	21 (30.4)	13 (18.8)	6 (8.7)	9 (13.0)	1 (1.4)	19 (27.5)
Mode of Payment	NHIF (75)	16 (21.3)	16 (21.3)	14 (18.7)	5 (6.7)	3 (4.0)	21 (28.0)
	OOP (67)	21 (31.3)	25 (37.3)	10 (14.9)	4 (6.0)	0	7 (10.4)
HF history	De novo HF (68)	18 (26.5)	21 (30.9)	11 (16.2)	7 (10.3)	3 (4.4)	8 (11.8)
	ADCHF (74)	19 (25.7)	20 (27.0)	13 (17.6)	2 (2.7)	0	20 (27.0)
Outcome at Discharge	Alive (115)	27 (23.5)	35 (30.4)	22 (19.1)	8 (7.0)	3 (2.6)	20 (17.4)
	Dead (27)	10 (37.0)	6 (22.2)	2 (7.4)	1 (3.7)	0	8 (29.6)
Vasopressors (32)		15 (40.5)	6 (14.6)	3 (12.5)	0	0	8 (28.6%)
**Comorbidities (N, %)**							
HIV (8)		1 (12.5)	5 (62.5)	0	2 (25.0)	0	0
HTN (45)		2 (4.4)	13 (28.9)	24 (53.3)	5 (11.1)	0	1 (2.2)
DM (17)		1 (5.9)	6 (35.3)	7 (41.2)	2 (11.8)	1 (5.9)	0
Chronic Lung Disease (31)		1 (3.2)	28 (90.3)	1 (3.2)	0	1 (3.2)	0
Liver Disease (47)		14 (29.8)	15 (31.9)	3 (6.4)	3 (6.4)	1 (2.1)	11 (23.4)
Renal Dysfunction (97)		33 (34.0)	22 (22.7)	22 (22.7)	7 (7.2)	1 (1.0)	12 (12.4)
Thyroid Dysfunction (26)		8 (30.8)	8 (30.8)	3 (11.5)	2 (7.7)	0	5 (19.2)
Dyslipidaemia (87)		21 (24.1)	30 (34.5)	16 (18.4)	7 (8.0)	2 (2.3)	11 (12.6)

Supplemental Table 6: Direct medical cost by the primary causes of HF

ADCHF, Acute decompensated congestive heart failure; CCU, Cardiac care unit; CM, Cardiomyopathy; CP, Cor pulmonale; DM, Diabetic mellitus; HF, Heart failure; HHD, hypertensive heart disease; HIV, Human immunodeficiency virus; HTN, Hypertension; IHD, Ischemic heart disease; NHIF, National Health Insurance Fund; OOP, Out-of-pocket payments; PD, Pericardial disease; RHD, Rheumatic heart disease; SD, Standard deviation.
